# Motivated Categories: Social Structures Shape the Construction of Social Categories Through Attentional Mechanisms

**DOI:** 10.1177/10888683231172255

**Published:** 2023-05-22

**Authors:** Suraiya Allidina, William A. Cunningham

**Affiliations:** 1 University of Toronto, Ontario, Canada

**Keywords:** social categorization, social structure, social hierarchies, attention

## Abstract

Social categorization is often framed as the antecedent to stereotyping, with perceivers rationally sorting the social world on the basis of perceptually salient categories before applying biased or motivated beliefs about those categories. Here, we instead suggest that the construction of social categories by individuals is itself subject to motivational influences, such that perceivers will attend to a given dimension of social categorization (e.g., race or gender) insofar as doing so fits within their motivations. Drawing from classic conceptualizations of social structure as the interplay of schemas and resources, we focus on how the motivations for shared schemas and for material benefits or resources may shape attention to social category dimensions. We outline the potential cognitive mechanisms through which these motivations may act on attention, before discussing the implications of this model for individual differences, conceptualizations of social categorization as rational information reduction, and prejudice reduction.

**Public Abstract:**

Social categories like race and gender often give rise to stereotypes and prejudice, and a great deal of research has focused on how motivations influence these biased beliefs. Here, we focus on potential biases in how these categories are even formed in the first place, suggesting that motivations can influence the very categories people use to group others. We propose that motivations to share schemas with other people and to gain resources shape people’s attention to dimensions like race, gender, and age in different contexts. Specifically, people will pay attention to dimensions to the degree that the conclusions produced from using those dimensions align with their motivations. Overall, we suggest that simply examining the downstream effects of social categorization like stereotyping and prejudice is not enough, and that research should look earlier in the process at how and when we form the categories on which those stereotypes are based.

Beginning with Lippmann’s adoption of the word “stereotype” to refer to the “pictures in our heads” (Lippmann, 1922), a long history of research efforts has aimed to understand how stereotypes form and how they might be reduced. Central to these efforts is the idea of social categorization, wherein perceivers sort the social world into groups of people and then form and apply beliefs about these groups, or stereotypes. Research within social cognition has classically framed social categorization as an antecedent to stereotyping (Dovidio & Gaertner, 2004), with research efforts focusing largely on how biased stereotypes are produced from the simple act of categorizing others into perceptually salient groups (Tajfel et al., 1971). A growing line of inquiry, for example, is focused on understanding how everyday cognitive processes or information processing strategies, which are regularly used and applied in non-social domains, can produce stereotypes and prejudice when applied in the social domain (Allidina & Cunningham, 2021; Bai et al., 2022; Gershman & Cikara, 2020; Hackel et al., 2022). From this perspective, categorization is typically seen as the solution to an information-reduction problem: since we cannot form individualized impressions of every person that we meet, we instead categorize people into groups based on statistical regularities to conserve cognitive resources (Macrae et al., 1994) and provide explanations of social behavior (Keil, 2006). Taken together, this work provides an answer to the question “why form categories”: social categories allow for both efficient prediction and lay explanation of human behavior. However, the related question of “why form *these* categories” has been relatively neglected, and is the focus of the current work.

Categories may be necessary for social prediction and explanation, but with a massive amount of social information available, how do perceivers settle on which features to use when grouping others into categories? Much of the work within social cognition simply assumes that perceivers group together individuals based on their most visually salient co-occuring features in a bottom-up manner. The categories that perceivers form are therefore taken as a given, and the downstream consequences of those categories are the main focus. This is not necessarily a problem for examining stereotyping and prejudice on the individual level, since much of the construction of categories occurs on a societal level, such that individuals within the same society generally have a shared set of commonly invoked social categories (Gelpi et al., 2022). It is therefore possible to examine an individual’s attitudes, beliefs, and behaviors toward culturally prominent groups without investigating the psychological reasons behind why the individual holds those categories. However, we argue that this approach misses a key piece of the puzzle in understanding societal or collective prejudice and inequality. Specifically, although this social construction may occur on a societal level, the effects of this construction are enacted by individual actors who internalize and eventually perpetuate these categories (Sewell, 1992). Thus, understanding how societally constructed categories are internalized and enacted by individuals may provide critical insights to our understanding of prejudice. In fact, Howard (1994) argues that by examining social categorizations, social cognition is uniquely positioned to address questions about how social structure and individual cognitions mutually sustain and reinforce each other, as these categorizations both internalize social structure and shape the actions that individuals take to alter structures. Understanding the psychological processes mediating this internalization and enactment may therefore be key to understanding the construction of categories.

## Outline of the Current Research

Categories may be used to make sense of social experience (an attempt to carve nature at its apparent joints), but the critical question here is why these categories, and why these joints? Given the constructed nature of most social categories, we suggest that perceivers flexibly shift their attention among different dimensions or ways of categorizing (e.g., race, gender, class) in different situations in a manner that fits with the existing schemas and narratives available to them. We propose that in this way, motivations can shape the category dimensions that people attend to in a way that often maintains social hierarchies and power structures. For example, perceivers may tend to pay more attention to race when doing so would reinforce structurally derived associations of White with positivity and Black with negativity, but downweight race when the pattern of racial categories and outcomes would challenge these associations. In other words, attention to a given dimension will increase when the conclusions that result from attending to that dimension align with existing structural narratives.

As social categories are situated within societal structures, the motivations most prominently shaping social attention may be those that arise from these structures. In line with the definition of structure as the mutual interplay of schemas and resources (Sewell, 1992), we outline how (a) motivations for shared reality with others and (b) motivations for the benefits that arise from social hierarchies can shape perceivers’ attention to potential category dimensions. Highlighting the shared and functional nature of categories, respectively, we propose that people will be motivated to form social categories that map onto the categories used by other perceivers in a given context and that produce or maintain outcomes that are beneficial to themselves.

As one example of this flexible shift in attention toward category dimensions, Hopkins and colleagues (1997) analyzed an interview with a police officer on the subject of police racism and found a strategic use of racial categorization at different points in the interview, with the officer highlighting race when it fit his narrative but instead down-weighting race when it did not. In the first part of the interview, the officer did not mention race at all, instead emphasizing the absence of intergroup conflict in the society and the police as trusted community members who serve the interests of society as a whole. However, when charges of racism are explicitly raised by the interviewer, the officer’s narrative shifts to highlighting the amount of interracial conflict in the society, positioning Black people as the instigators and the “West Indian way of life” as the problem. As this example illustrates, conceptualizing categorization as a flexible and strategic process leads to a slightly different focus of inquiry: instead of examining how perceivers go from rational, obvious, or perceptually salient group divisions to biased beliefs and behaviors toward those groups, we can instead look at how initial group divisions may be biased in the first place in a manner that readily produces the resulting stereotypes and justifies the ensuing behaviors.

The remainder of this paper proceeds as follows. In section 2, “Background: The Construction of Categories Along a Single Dimension” we provide some background on the construction of categories along a single dimension (such as race or gender), both on a collective level and an individual level. In section 3, “Category Construction Through the Direction of Attention” we describe how perceivers’ motivations may shape their attention among different potential category dimensions, thus situationally constructing categories in a motivationally congruent manner. Section 4, “Socio-Structural Motivations and Attention to Category Dimensions” discusses the motivations that arise from social structures and how these motivations may shape the categorization process, focusing on the selection of dimensions. In section 5, “What Are the Potential Cognitive Mechanisms Through Which This Occurs?” we draw from the literature on category learning and selective attention to outline potential mechanisms through which this structural influence may shape social categorization. Finally, sections 6-8, “What Implications Does This Have for Individual Differences in Category Structures?” “How Does This Fit With the Idea of a ‘Rational Actor’ or the Utility of Social Categorization as Information Reduction?” and “What Implications Does This Have for Reducing Prejudice and Dismantling Structures?” discuss the implications of this model for a variety of domains, including individual differences in category structures, conceptualizations of perceivers as “rational actors,” and prejudice reduction. In reviewing the work in the remainder of this paper, we acknowledge that much of the research suffers from the same limitations that the field of psychology as a whole is struggling with—namely a lack of diversity in samples that limits generalizability. Thus, we note that applicability to non-WEIRD (Western, Educated, Industralized, Rich, and Democratic; Henrich et al., 2010) samples may suffer until more research is done to examine these processes in more heterogeneous populations. Throughout this work, we apply a psychological lens to understanding the modern construction of social categories. Thus, we focus not on how different categories came to be prominent in different modern and historical societies (which has been detailed in work from other fields), but on how individuals flexibly adopt and attend to different category dimensions within the societal constraints that exist.

## Background: The Construction of Categories Along a Single Dimension

The social world presents perceivers with a vast array of visual information, from which we produce conceptualizations of dimensions like race and gender. The information we use to create these dimensions, like most of the visual information we are confronted with, is largely continuous; that is, categorical structure is not inherent to things like race and gender. Faced with all this continuous visual information, we learn to selectively discretize certain dimensions (e.g., race) into categories, which then come to have particular meanings and locations within society attached to them. The act of categorizing something into discrete groups, even if no additional information is gained from translating this continuous information into categories, shapes the way the information is used in social perception and judgment (e.g., Yamauchi, 2005). The dimension of age serves as an illuminating example of an inherently continuous dimension that is discretized into categorical terms, which then come to have different social and legal meanings attached to them (which can differ across cultures; Barak, 2009). For example, terms that represent age categories like “child” or “adolescent” carry implications of immaturity or incomplete development, and these categories come with a strong system of power relations (Mintz, 2007). The differences between a 17-year-old and a 19-year-old may in actuality be small, but these two individuals have vastly different social and legal statuses in societies where 18 is considered the start of adulthood.

Work from the constructionist perspective has focused on how our social realities are actively constructed and given meaning in everyday life. The societal construction of social categories has received a great deal of attention across fields, with work detailing the social and structural influences both on how these dimensions come to exist in the first place (Cosmides et al., 2003; Golash-Boza, 2016; Machery & Faucher, 2005; Pietraszewski et al., 2014) and on how certain groups become located at different points on these dimensions (e.g., changing conceptions of who is classified as “White”; Chaney et al., 2021; Ignatiev, 1994; Roediger, 2006). For example, the concept of “doing gender” suggests that gender categories are societally constructed through everyday routines and behaviors that both emerge from and reinforce these categories (West & Zimmerman, 1987). The social construction of race has also been well established, with some scholars positing that our modern concept of “race” in North America emerged out of slavery around the end of the 17th century (Allen, 1994). As these examples demonstrate, much of this construction occurs on a societal level, in that categories are collectively constructed and reified through interactions among agents. For categories like “race,” “gender,” and even “slave” to have utility, individuals must, therefore, use the meanings that these categories have been ascribed by society.

Categories may be imbued with meaning largely on a societal level, but individuals still have some power to shape the construction of the social categories they use. Work within psychology has detailed how the construction of categories *within* a given dimension (e.g., race or gender) occurs within individuals. A growing body of research has examined how categorization along continuous dimensions can be biased on the individual level in a way that typically serves to maintain dominant or structural narratives about different groups (Caruso et al., 2009; Castano et al., 2002; Freeman et al., 2011; Hugenberg & Bodenhausen, 2004; Peery & Bodenhausen, 2008; Penner & Saperstein, 2008; Plaks et al., 2012; Richeson & Trawalter, 2005; Saperstein & Penner, 2012; Stelzl et al., 2008). The majority of this research focuses on racial categorization. Caruso and colleagues (2009), for example, found that biracial political candidates were seen as lighter skin-toned when their partisanship matched the participant’s, but as darker-toned when they had the opposite partisanship. Similarly, an athlete with dual Canadian and Jamaican identities was described by Canadian newspapers primarily as Canadian after he won the gold medal, but predominantly as Jamaican after being disqualified (Stelzl et al., 2008). This fluidity in racial categorization is also shaped by status, with perceived status influencing both how perceivers categorize a racially ambiguous face (Freeman et al., 2011) and even which racial groups people self-identify with (Penner & Saperstein, 2008; Saperstein & Penner, 2012). Overall, this work suggests that the individual-level process of racial categorization is itself subject to motivational influences, especially when the target of categorization is somewhat racially ambiguous.

Rather than simply reflecting motivational influences on the application of stereotypes to social categories, these studies point to top-down effects on the creation of the categories themselves, which occurs earlier than the application of stereotypes (e.g., Ito & Tomelleri, 2017; Zhang et al., 2018). Studies employing a variety of techniques to investigate early timescales of person perception have supported the temporal primacy of these effects. For example, top-down effects on the selection of category boundaries occur even in speeded tasks that allow little time for deliberation (e.g., Peery & Bodenhausen, 2008). Work from Freeman and colleagues (e.g., Freeman & Ambady, 2010, 2011; Freeman et al., 2013; Hehman et al., 2015) using a combination of mouse-tracking experiments and simulations provides further evidence that categorization itself is subject to top-down influences. By tracking participants’ mouse trajectories while they categorize a target into one of two social groups (e.g., male or female), the relative activation of unchosen categories can be assessed at high temporal granularity. Results from many such studies indicate that top-down effects on categorization along a dimension like race or gender emerge early and influence subsequent processing (Freeman & Ambady, 2010, 2011; Freeman et al., 2013; Hehman et al., 2015). Evidence from electroencephalography studies has similarly provided evidence that motivations can influence very early neural responses to faces of different races or religions (Cunningham et al., 2012; Derks et al., 2015). Taken together, these diverse lines of work provide converging evidence for top-down influences on social categorization that occur at very early timescales in the process of person perception.

## Category Construction Through the Direction of Attention

**Figure 1.**
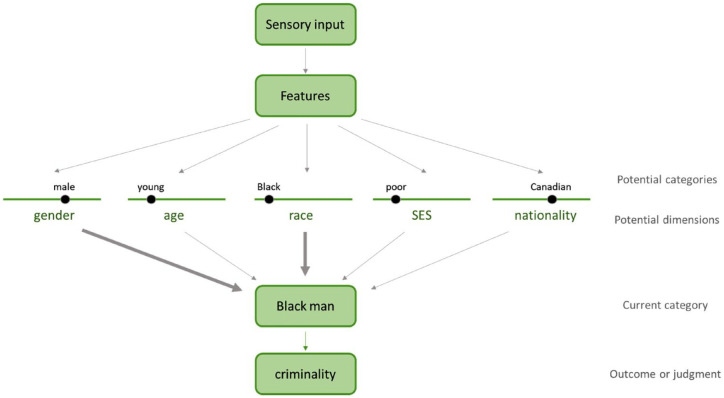
Illustration of dimension weighting in social categorization. *Note.* This model outlines how perceivers go from receiving sensory input about a target to making a judgment about the target by way of social categories. Perceivers use a target’s features to place them along a variety of potential dimensions, which are largely societally determined. These dimensions are then differentially weighted to produce a categorical conception of the target that the perceiver uses to make judgments and predict relevant outcomes. Structurally derived motivations aim to maintain the link between a particular outcome (here, criminality) and a potential category (e.g., Black). To do so, these motivations can act on various stages of this process, including the potential categories and dimensions that are even considered and tested, the definition and scope of the outcome of interest, and the perceived similarity or typicality of the target in relation to various potential categories. Rather than being a purely sequential process, the selection and weighting of dimensions can also influence the way features are translated into potential categories.

The research reviewed above demonstrates that motivational influences can shape where a particular target is placed on a single dimension of categorization (e.g., race), but a relatively neglected aspect of study has been the *selection* of dimensions along which categories are formed. In any given situation, there is an almost endless number of ways to potentially carve up the social world, including both commonly studied categories like race, gender, and age as well as other information like occupation, social network, and ideological beliefs. Although categories are largely created and imbued with meaning on a societal level, each situation requires an individual to attend to (and thereby deem relevant) certain dimensions while ignoring others. In other words, features are given meaning on a societal level by being discretized into categories but can be given meaning or weight on an individual or situational level by being attended to. The process outlined in Figure 1 demonstrates this distinction: features (which are formed from sensory input) are used to place targets along a variety of potential dimensions, which are then weighted to form a current conceptualization of the target. For example, the feature of dark skin may be used to place somebody into the category “Black,” whereas the features of broad shoulders and short hair may be used to place the same person into the category “male.” Both the potential dimensions (race, gender) and the specific categories located on these dimensions (Black, male) are largely a product of current and historical societal forces, reviewed elsewhere (Lorber & Farrell, 1991; Smedley & Smedley, 2005).

However, this is not the end of the categorization process, as a perceiver cannot conceivably use *all* the potential dimensions available to them to form a judgment about the target. Doing so may be akin to forming an individualized impression of the target, which often is not feasible within the relatively short time frames of most social impression formation. Instead, the perceiver weights and combines these different dimensions to produce a categorical conception of the target (labeled “current category” in Figure 1), which they can then use to form judgments or predict outcomes about the target. In this example, the perceiver may more heavily weigh the dimensions of race and gender than alternative dimensions like age, socioeconomic status, or nationality, and end up with a conceptualization of the target as a “Black man.” This conceptualization will then shape the perceiver’s judgments or predictions about the relevant outcome; in this case, the conception of the target as a Black man will likely stereotypically increase perceptions of him as a criminal. The contextual selection of dimensions is thoroughly described by Petsko and colleagues (2022), who propose that people have a repertoire of “lenses” for categorizing others that are flexibly adopted based on the perceiver and context. In line with the lens model, the weighting of dimensions outlined in Figure 1 can occur both on single dimensions (e.g., race) or an intersection of dimensions (e.g., race and gender), each of which gives rise to its own set of stereotypes (Petsko et al., 2022).

Much of the existing work has largely focused on the upper half of this model, examining how perceivers use a target’s features to place them along various potential dimensions (which are largely societally produced). Here, we instead focus on the motivational forces shaping how perceivers weigh and combine dimensions to produce categorical conceptualizations of a target, which they then use to make judgments and decisions. In this example, placing higher weight on race and gender led to a very different outcome than would weighting alternative dimensions such as age and nationality; conceptualizing the same target as a “young Canadian” would not have produced the same stereotypes as conceptualizing him as a “Black man.” When predicting a stereotypical outcome like criminality, however, the latter categorization is much more likely than the former; we focus here on why that is. Building on the work detailing the social construction of categories on a structural level, we outline how motivations for shared schemas and for resources or self-benefit may influence which dimensions are selectively recruited and attended to on the individual level.

Existing work on motivational influences on dimensional attention typically falls into one of two categories. The first focuses on self-image maintenance as the motivation, investigating how people’s desire to preserve or lift up their self-esteem may shape the category dimensions they use (Fein & Spencer, 1997; Mussweiler et al., 2000; Sinclair & Kunda, 1999). For example, Fein and Spencer (1997) found that experimentally threatening someone’s self-concept increases the likelihood that they use a negatively stereotyped dimension to categorize a target. The second category of research takes an evolutionary psychology approach to examining motivated attention to social category dimensions (Kenrick et al., 2010; Maner et al., 2008, 2012). In particular, the fundamental social motive perspective (Kenrick et al., 2010; Neel et al., 2016) posits a set of evolutionarily rooted goals that, when active, shape aspects of memory, attention, and social inference in functionally specific ways. This framework suggests that perceivers will categorize others using the traits that are most relevant to their currently active motive, which may include things like mate selection, disease avoidance, or self-protection. For example, Maner and colleagues (2012) found that a mate-searching prime leads participants to categorize other-gender targets by attractiveness, whereas a self-protection prime instead leads to categorization by race. Closely related is the affordance management approach to stereotypes (Neuberg et al., 2020), which posits that people are oriented toward the opportunities and threats afforded by others. Under this approach, stereotypes function to provide information about these affordances that perceivers can use to maximize opportunities and minimize threats. Thus, the framework suggests that people should attend to the category dimensions that are most informative about others’ affordance-implying behaviors.

The model we propose here is compatible with many parts of the fundamental motives and affordance management approaches,^1^ focusing on slightly different levels of analysis. If, as the affordance management approach suggests, people are oriented toward the affordances provided by others, our model is aimed at understanding *why* particular groups are stereotyped as affording particular opportunities and threats. In contrast to the evolutionary motives outlined by the perspectives discussed above, the structural motivations we outline here are closely linked with the current social order, acting as a potential mediator in the effect of social structures on cognition. We therefore suggest that structural motivations for shared schemas and resources (described in the following section) can help explain how these more “fundamental” motivations might materialize. For example, while the fundamental motive perspective posits that we are motivated to find mates and avoid disease, the structural motivations we describe may help shape who is considered a good mate and who is thought to carry disease. Thus, our model aims to complement existing work on the role of motivations in social categorization by bringing a structural lens to motivated cognition.

In this work, we propose that motivations to maintain current social structures (because of the shared schemas and material resources they provide) can shape the dimensions that perceivers attend to in a way that upholds the social structure producing these motivations. As demonstrated in Figure 1 and explained further in the following sections, these motivations aim to maintain the link between a potential category (e.g., “Black”) and a particular judgment or outcome (e.g., “criminality”). Given the socially constructed nature of the categories perceivers tend to rely on, we suggest that perceivers adopt a flexible differential focus on dimensions such as race, gender, and class in different situations in a manner that fits with the existing structural schemas and narratives available to them. For example, this differential focus may take the form of attending to race when doing so would reinforce positive associations with White people and negative associations with Black people, but down-weighting race when racial patterns challenge these associations. In other words, when attending to a given dimension produces conclusions that align with existing structural narratives, perceivers will be more likely to pay attention to that dimension.

Motivations may not only shift attention laterally between different dimensions at a basic level of categorization (e.g., race or gender) but also to subordinate categories that involve an intersection of dimensions (e.g., Black man or white woman). It may be useful at some times for a White perceiver to think of a target as “Black,” whereas at other times it may be more useful to think of them as a “Black woman” or a “Black female academic.” This is in line with the lens model of intersectional stereotyping (Petsko et al., 2022), which proposes that lenses can be either singular (e.g., categorizing by race) or more complex (e.g., categorizing at the intersection of race and gender), giving rise to different sets of stereotypes. Here, we build on this model by outlining how the perceiver’s motivations can shape which “lens” they adopt when categorizing others. The level at which a perceiver categorizes someone may thus depend not only on how rich their representation of the target is but also on the stereotypes or societal positions of the various potential categories that could be applied. For example, someone motivated to highlight the threatening nature of a Black target may be more likely to categorize the target only by race if the target is a woman, but by both race and gender if the target is a male. This is because the category “Black” may elicit more threat-related stereotypes than the category “Black woman,” but less threat-related stereotypes than the category “Black man.” If, however, the perceiver was motivated to highlight stereotypes related to welfare, the opposite pattern may emerge. Related to this notion, a great deal of prior work has demonstrated that people “subtype” group members who disconfirm stereotypes into a separate subcategory to maintain existing beliefs about the group as a whole (Devine & Baker, 1991; Johnston & Hewstone, 1992; Maurer et al., 1995; Richards & Hewstone, 2001). Building on this work, people may more generally form subgroups or not depending on how well the potential subgroups fit within their existing beliefs and narratives. In other words, motivations may shape not only the selection of single dimensions but also the selective creation of subcategories.

The idea of cognitive resource management is still central to this perspective, but we approach the idea of such cognitive efficiency from a slightly different angle. In line with classic accounts of social perception, cognitive effort and resource capacities place major constraints on our abilities to form individualized person impressions. When navigating the social world, people therefore form categories to save on cognitive resources, and in doing so will need to identify the features that are most relevant for use in category formation. According to theories on relevance, the greater the effort to process some cognitive input, the less rewarding that input will be to process, and hence the less relevant or deserving of attention it is (Wilson & Sperber, 2002). Applying this idea, classic approaches suggest that the things that are less effortful to process (or more salient) will therefore be deemed more relevant (e.g., people pay so much attention to race because racial differences are so visually salient).

Here, we focus on the ways in which we set up our schemas in such a way to *make* certain things less effortful to process, by making us more automatically attuned to them. This increase in the ease of processing certain features actually makes these features more relevant. This is not necessarily in contrast with the classic ways of conceptualizing relevance, as both processes are likely to occur to some extent, but it shifts the focus of inquiry to the construction of categories rather than simply their application. For example, whereas classic approaches may say that perceivers are highly attuned to gender because it is often very easy to process or salient, we instead focus on the ways in which we *make* gender easy to process because it is relevant to us. Research on artificially created groups has demonstrated an increase in processing as a result of induced relevance, with perceivers differentially processing information about various groups along a dimension according to how motivated they are for that dimension to be relevant. Work from Van Bavel and Cunningham (2009, 2012), for example, demonstrates that the motivation to attend to one dimension (arbitrary minimal groups created in the context of the experiment) over another dimension (race) makes the motivationally congruent dimension more memorable and even shapes relatively automatic racial biases. More generally, the centrality of the ingroup in social perception may suggest that we can more easily process those we deem relevant to us (Brewer, 1979, 1999). This idea has been widely studied in artificial groups created within the experimental context but applies more broadly to social groups in general. By making salient those things that can most readily fit into our existing or desired state of the world, our schemas and structures construct relevance.

## Socio-Structural Motivations and Attention to Category Dimensions

If individuals situationally construct social categories in a way that fits with their motivations, understanding social categorization requires an understanding of the motivational factors that shape an individual’s attention to the different dimensions that could be used in creating categories. Since categories are fundamentally tied up in societal structure, structurally derived motivations are likely to play a prominent role in shaping the use of social categories (although more individual-level motivations can play a role as well). We focus here on two main categories of motivations that societal structures impart onto individuals: the motivation for shared schemas and narratives and the motivation for resources.

### Motivations for Shared Schemas

First, perceivers will adopt category structures that align with those of others and fit within shared structural narratives to facilitate social prediction, communication, and interaction. Shared reality theory (Echterhoff et al., 2009; Hardin & Higgins, 1996) highlights the pervasive motive to have a shared reality with others, involving motivated common inner states with other people about some target in the world. Achieving such a sense of shared reality allows people to fulfill both epistemic and relational needs and is touted as a “potentially important everyday mechanism underlying the construction of culturally shared memories and evaluations of the world” (Echterhoff et al., 2009, p. 515). This drive for shared reality constrains our goals in predicting the world, such that individuals are trying to minimize prediction errors not just individually, but inter-individually (Wheeler et al., 2020). Through this socially constrained error minimization, our contexts, cultures, and societies shape what we deem relevant, leading to a kind of shared attention (Ramstead et al., 2016; Veissière et al., 2019). The nature of these schemas is such that they shape cognition and behavior while remaining invisible to the perceiver; they are, in a sense, the frame through which we see the world.

Applying this desire for shared reality to categorization, people will be motivated to have mental categories of other people that map onto the categories used by others in their society. No matter how efficiently an individual’s categories condense social information, the fundamentally shared nature of such categories means that they will only have utility insofar as they map onto categories that are simultaneously adopted by others with whom they are attempting to interact (Echterhoff et al., 2009; Hardin & Higgins, 1996; Howard, 1994; Veissière et al., 2019; Wheeler et al., 2020). No matter how salient hair color is to a given perceiver, making generalizations about brown-haired versus red-haired individuals will usually not carry the same communicative weight as will generalizing about Black versus White people, or men versus women. Perceivers will, therefore, adopt category structures that tend not to challenge shared narratives or assumptions, since these shared assumptions are necessary for communication and prediction (Jost et al., 2008).

### Motivations for Resources

The second category of relevant motivations concerns the *material outcomes* that our shared categories produce on a societal level: perceivers will be motivated toward category structures that are beneficial for them, in the sense that they confer material or non-material benefits. In particular, those in dominant groups will be likely to adopt category structures that maintain current structural schemas because of the resources and power conferred to them by the current social order.

Whereas work within social psychology tends to localize prejudice and discrimination within the individual, work in sociology and other fields instead positions racism as a primarily structural phenomenon that centers on domination (Bonilla-Silva, 2015; Desmond & Emirbayer, 2009; Golash-Boza, 2016; Reicher & Hopkins, 2001). Under the materialist framework put forth by Bonilla-Silva, for example, domination is central to racial ideology, with prejudice framed as the ideological expression of dominance (and therefore a collective rather than individual phenomenon). Racism under this framework is “rational”: individuals support or resist racial structures because doing so is beneficial to them (or they believe so). A prime example of this is provided by Roithmayr (2014), who describes the racial cartels of the Jim Crow era, focusing on “the material benefits to collective action that monopolize benefits for one group at the expense of another” (p. 28). Examples of such cartel conduct, which often functioned with government support, ranged from White homeowner associations that engaged in housing discrimination to maintain their own wealth and property value to White unions that excluded Black workers from organizing to keep them from earning the high wages associated with the profession.

Integrating these sociological perspectives on racial domination with psychological processes is social dominance theory (Pratto et al., 1994), which highlights the role of “legitimizing myths,” or shared societal ideologies, in reproducing dominance hierarchies. In this theory, hierarchy-enhancing beliefs within members of dominant groups (as well as other groups, although to a lesser extent) function to maintain the groups’ privileged positions, in essence maintaining their unequal share of resources. Social dominance theory outlines three sets of group-based social hierarchies: those based on age, those based on gender, and those referred to as “arbitrary-set,” which represent socially constructed categories that vary across cultures and include things like race, caste, class, and religion. Throughout both of these frameworks, the social construction of race and other social categories is key, with Bonilla-Silva in particular highlighting that racial categories are produced by systems of domination, rather than the other way around.

A critical point here is that categories are not simply cognitive constructs divorced from the material world. Rather, categories and stereotypes are functional: they do not just represent social reality but are used to explain and justify the treatment of others (Allport, 1954; Dixon, 2017; Jost & Banaji, 1994; J. C. Phelan et al., 2008), including the distribution of material resources. As such, categories are closely intertwined with distributions of resources and benefits, and motivations for resources may therefore shape the construction of social categories that an individual uses.

### “Structure” as the Interplay of Schemas and Resources

With these two types of motivations in mind, we turn to classic definitions of “structure” from the sociological literature. In particular, we draw on the definition proposed by Sewell (1992), who builds on the classic definition put forth by Giddens (1979). Specifically, Sewell conceptualizes social structure as comprising two main parts: *schemas*, which are informal generalizable procedures and assumptions that are often unconscious, and *resources*, which can be both human (e.g., knowledge, commitments) and nonhuman (e.g., money, land). These two aspects parallel the two categories of motivations discussed above, with schemas forming the basis for shared reality with others and resources representing the material benefits that are both produced by and reinforce category structures. Under this definition, schemas and resources mutually constrain and inform each other, such that one cannot exist for long without the other. Schemas direct the use of resources; for example, money would be useless without the shared understanding and acceptance that it can be traded for goods. Similarly, resources can be “read” to recover the schemas they instantiate. Together, schemas and resources shape and constrain social action, while also being reproduced by that action.

To further illustrate this definition of structure, Lewis (2004) shows the interdependent nature of schemas and resources in the context of “White flight.” She argues that White people’s schemas consist of stereotypes that make them prefer living in White neighborhoods to comparable Black neighborhoods, and as a result of these schemas, the housing prices in White neighborhoods become inflated. Thus, the schemas create resource outcomes or material benefits for White people, and these resources in turn appear to confirm the schemas (i.e., White neighborhoods are nicer and Black neighborhoods are worse). Critically, this cycle of schemas and resources tends to be reproduced even without individual agents’ awareness or intention (though not necessarily automatically). That is, while we refer to structural “motivations,” these motivations need not be intentional or explicit to reproduce structures. The question here, then, is how these schemas function at the level of categorization to constrain social action and further reinforce the resource distributions that help produce them.

In this work, we aim to apply this conceptualization of structure to the domain of social categorization, examining how structurally produced motivations (i.e., motivations for shared schemas and for resources) may shape the production of social categories in individuals through attention. Since categories form part of the shared cultural schemas that underpin and are reflected in resources, Howard (1994) argues that categorization is primarily an inter-individual phenomenon, functioning to both internalize structure and act as the vehicle through which structure is enacted in the world. Using insights from shared reality theory, we elaborate on this idea by examining how the drive toward shared schemas may shape the social categories that perceivers adopt and use by directing their attention to particular dimensions. Although previous work has demonstrated that perceived consensus can influence one’s stereotypes about a group (Sechrist & Stangor, 2001; Stangor et al., 2001), we extend this to argue that these shared schemas shape the very groups that are formed in the first place.

Furthermore, building on social dominance theory, we ask how dominance-reproducing ideologies may act specifically on categorization processes to reinforce hierarchies. In other words, how might the drive toward maintaining unequal resource distributions shape the formation of the categories that comprise these hierarchies? Again, such a drive need not be intentional or explicit; rather, this motivation may alter the assumptions that shape the way we interact with the world, giving rise to some of the ideologies and beliefs that legitimize hierarchies and maintain the status quo. Based on the notion that racism is reproduced in our everyday worlds through day-to-day practices and actions (Roithmayr, 2014; Salter et al., 2018), we aim to examine how this framing specifically shapes the category and feature dimensions that we attend to in a manner that instantiates and reproduces structures. In examining this question, our goal is to integrate levels of analysis by more fully bringing structurally embedded notions of domination and power into the study of psychological processes. In doing so, we hope to contribute to a growing body of calls from other researchers to incorporate more structural and historical aspects to the psychological study of race and other types of social groups (Kraus & Torrez, 2020; Oishi & Graham, 2010; Salter & Adams, 2013; Salter et al., 2018; Tileagă, 2017; Trawalter et al., 2020).

Although we believe motivations for schemas and resources are particularly relevant mediators in the effect of social structures on categorization, these are of course not the only motivations relevant to social cognition. Rather, motivations for schemas and resources may act alongside other relevant goals, such as the motivations to maintain a positive self-image (Tajfel & Turner, 1986) or be non-prejudiced (Plant & Devine, 1998). Higher-level motivations for schemas and resources may constrain the fulfillment of other goals, and in some cases could even be responsible for other motivations. For example, motivations for group-based self-esteem could in some instances be reinterpreted as motivations to maintain the positive shared within-group schema. However, the precise relationships among these motivations remain an open question for future work. Our framework, therefore, rests on the premises that (a) motivations for schemas and resources exist, (b) these motivations shape attention to category dimensions, and (c) these motivations act over and above other well-established motivations *or* explain those other motivations.

## What Are the Potential Cognitive Mechanisms Through Which This Occurs?

**Figure 2.**
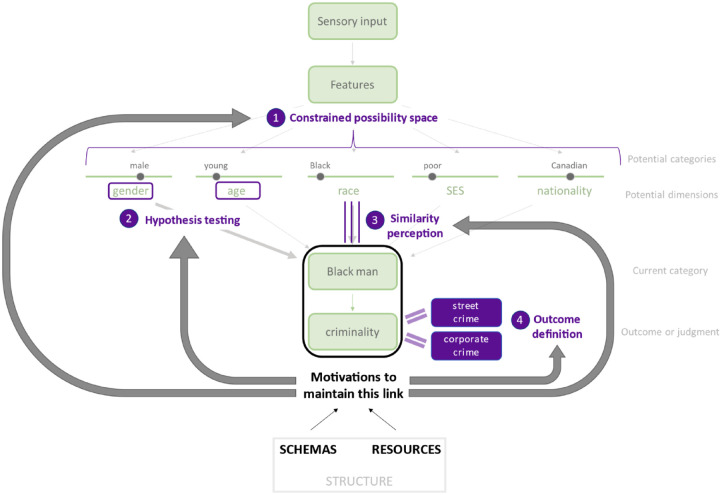
Mechanisms Through Which Motivations for Shared Schemas and Resources May Act on the Selection of Dimensions to Produce Categories *Note.* (1) Motivations may constrain the possibility space of potential categories, shaping which categories are even considered by the perceiver as being potentially relevant. (2) Motivations may determine the hypotheses that perceivers seek to test, such that perceivers first test dimensions that would produce desired conclusions before those that would produce undesired conclusions. (3) Motivations may alter the perception of similarity between a particular target and an overall category, such that perceptions of similarity are increased for motivationally congruent outcomes and decreased for motivationally incongruent targets. (4) Motivations may shape the selection and definition of outcomes, such that outcomes are defined in a manner that maintains existing psychological links between the outcome and the category.

The process outlined in Figure 1 suggests a number of potential places where motivations can influence categorizations. In line with the focus of this paper, we outline several mechanisms through which structurally derived motivations may specifically influence the weighting of different potential category dimensions. As outlined in Figure 2, motivations may act on the potential dimensions and categories that are considered and tested (through *constrained possibility spaces* and *hypothesis testing*), the perceived relation of the target to the various potential categories (through *similarity perception*), and the definition and scope of the outcome to be predicted (through *outcome selection*).

### Constrained Possibility Spaces

First, structures can constrain the possibility space entertained by the perceiver (Haslanger, 2016). A key part of structures’ power lies in their invisibility: structural schemas shape the everyday assumptions we make about people and power in a way that is often invisible to us, since they color all of social (and other) perception in a way that makes it difficult to “step outside” our schemas and critically examine them. Beliefs that in reality arise from these schemas may be considered “default” or “neutral,” such that they are maintained through the illusion of objectivity: the perceiver doesn’t even consider anything else as being possible. Lewis (2004) describes this as “the ‘of course’ way of understanding social existence,” with hegemony as a system that manages to “occupy the empty space of ‘normality’ in our culture” (p. 632).

In the domain of social categorization, this constrained possibility space can take the form of shaping the potential dimensions and categories that are even considered by a perceiver, which are then seen as neutral or objective. Work on motivated reasoning often adopts a Bayesian reasoning frame, wherein prior beliefs are updated by the likelihood (data) to form posterior beliefs (Gershman, 2019; M. Kim et al., 2020). In this way, researchers can examine whether belief updating is “rational” or not. In this framework, if the prior rules out some possibilities, they cannot be “rescued” by the likelihood, such that no amount of data can lead someone to a conclusion or categorization that they have a priori decided is not true. Thus, the initial possibility space entertained by the perceiver is critical. Dominant schemas may place high prior weight on a certain dimension (e.g., shared schemas generally say that race is a more important dimension than hair color) or high prior weight on a certain conclusion, which then shapes attention to the relevant dimension (e.g., shared schemas may say that White people excel academically while Black people excel athletically, so perceivers may attend to race in a context-dependent way that is congruent with these expectations).

### Hypothesis Testing

Within this constrained possibility space, structural schemas may also determine the “hypotheses” that perceivers seek to test, and bias credit assignment to these hypotheses in a self-maintaining way. Hypothesis generation and testing is central to learning: when presented with multiple potential cues or dimensions that could be responsible for an outcome, perceivers go through a process of hypothesis testing where they select a cue, subject it to a hypothesis test, and then use feedback to determine if the hypothesis was correct or incorrect (Akaishi et al., 2016). If the hypothesis was correct, the selected cue is given credit for the outcome; if it was incorrect, the cue is not given credit or is given less credit for the outcome. In social category learning where motivational influences are common, this credit assignment may occur in a biased fashion. Indeed, Pyszczynski and Greenberg (1987) outline a biased hypothesis-testing model of how motivations influence information processing. Focusing primarily on self-esteem motivations, they describe how motivations can act at various stages of the hypothesis generation and testing procedure to help the perceiver reach desired conclusions while maintaining an illusion of objectivity.

Applying the logic of biased hypothesis testing to social category learning, perceivers may first test hypotheses that are in line with their motivations (and if supported, fail to test further hypotheses; Nickerson, 1998; Smith et al., 2008; Wason, 1968). Furthermore, if outcomes are probabilistic, a disconfirmation on an undesirable cue may carry more weight than a disconfirmation on a desirable cue. In other words, people may have higher learning rates for schema-congruent information or may make use of discounting strategies to disregard incongruent information. In fact, the hypotheses that are tested in part influence the credit that is assigned: when subjects received feedback that they were correct, they attributed credit almost exclusively to the cue they had selected as the focus of their current hypothesis test; when they received feedback that they were incorrect, however, they still attributed around 40% of the credit to the cue they had selected for the test (even though it led to the wrong prediction; Akaishi et al., 2016). Thus, even selecting a dimension as the basis for a test increases the likelihood of assigning credit to that dimension, even if it fails to predict the outcome accurately. A perceiver who hypothesizes that race predicts intelligence, for example, may assign some credit to their hypothesis just by virtue of having tested it even if it is disproven by the data.

The specific hypotheses that are tested first may differ depending on the particular motivations and schemas of a given perceiver. Some forms of anti-Black racism, for example, take the form of treating Black people as members of an unintelligent but highly physical race. People who hold this kind of ideology would likely test racial hypotheses in both intelligence and athletic contexts since attending to race in these contexts could support their narrative. Other kinds of anti-Blackness simply view Black people as inferior in all or most domains; perceivers who hold this kind of view would be less likely to test hypotheses about race in athletic contexts, since doing so may lead to the counter-narrative conclusion that Black people are better at athletics than White people. Importantly, however, even a perceiver who does not have the specific motivation to believe that, for example, White people are good at academics and Black people are good at athletics is likely to still adopt some form of these attentional allocations. The bulk of the motivational “work” may be done by the historical creation of societal schemas, and so simply following societal norms on when race is relevant and when it is not can further reinforce hierarchies even without any explicit intentions to do so on the part of the perceiver.

### Similarity Perception

Structures may also shape attention to dimensions in a schema-congruent manner through biasing the perceived similarity of the target to the various potential categories. Similarity is central to categorization, shaping both the groupings that are formed and the dimensions that are highlighted or de-weighted when forming those groupings. As such, perceived similarity governs attention updating in many cognitive models of categorization (e.g., Kruschke, 1992; Love et al., 2004), which represent attention as a series of weights applied to each dimension (e.g., shape, size, color). In these models, similarity is represented by distance in psychological space, with similar items close together and dissimilar items further apart. Attention to a given dimension is thus updated as a function of the distance between a presented stimulus item and the existing category that it is classified into: perceivers will attend to a dimension more if the stimulus is similar to the existing category along that dimension, and will attend less if the stimulus is different from the existing category on that dimension.

One way that structural or motivational influences may affect attention, therefore, is by influencing perceptions of similarity. In line with work demonstrating that prior knowledge can affect category learning through altered similarity perception (Sun & Yin, 2020; Vandierendonck & Rosseel, 2000), we suggest that perceptions of how similar a stimulus is to an existing category on that dimension may become inflated or deflated in line with the perceiver’s schemas and motivations. Although dimensions like race are generally viewed as categorical, perceivers still notice and acknowledge variation in prototypicality within these categories. For example, darker-skinned Black people, who are seen as more prototypical of the category “Black,” face even more discrimination than lighter-skinned Black people (Uzogara & Jackson, 2016), even if both are categorized as Black. These perceptions of prototypicality shape similarity judgments and may therefore be the target of motivational factors. In line with this idea, perceptions of Black targets’ skin tone shifts according to how well-liked they are: perceivers see liked Black people as lighter-toned than disliked Black people (Caruso et al., 2009). In other words, schemas that position Black people as negative shape the perceived similarity between a given target and the category “Black” on racial dimensions. This altered similarity perception may then influence subsequent attention to that dimension: viewing liked Black people as lighter-toned positions them as farther away from the “Black” prototype, increasing distance between the target and the category and therefore decreasing attention to race. Similarly, disliked Black people are seen as darker-toned and therefore closer to the Black category prototype, decreasing distance on that dimension and therefore increasing attention to race for disliked Black exemplars. In this manner, attention to race may be de-weighted for positive Black exemplars and increased for negative Black exemplars, thereby further reinforcing negative associations with Black people. For example, Black people who do poorly in academics may be seen as more similar to the group “Black people” than those who do well academically, and vice versa for White people, such that attention to race is increased in stereotypic ways when predicting academic outcomes. This altered similarity space could occur either through subtyping, in which non-prototypical group members are put into their own subgroup to avoid updating impressions of the rest of the group (Richards & Hewstone, 2001), or simply through stretched or compressed similarity spaces. Indeed, since similarity perceptions are largely subjective, changing perceptions of similarity in such a way can allow for schema-consistent allocations of attention while still maintaining “rationality.” Motivations may be especially likely to play a role in cases where group positions are somewhat more ambiguous, as in the case of racial classification of Latino people or gender classification of gender non-conforming individuals.

### Outcome Selection

In addition to shaping the dimensions that are considered and tested, structural schemas can also shape the outcomes or behaviors of interest that are attended to. Many cognitive models of categorization assume that the match between outcomes and the predictions generated by category membership determines how attention is allocated to category dimensions. If a given dimension generates predictions that are consistent with the outcome, that dimension will receive more attention in the future, whereas dimensions that produce incorrect predictions will be downweighted. However, in most category models, the scope of these outcomes is fixed and entered into the model by the experimenter, with the selection of relevant outcomes unaddressed (but see Guest & Love, 2019). In the social world, by contrast, there is often a great deal of flexibility in how these outcomes are selected or defined: someone whose goal is to hire the best candidate for a job can define “best” in any number of ways (e.g., highest standardized test scores, most relevant previous experience, highest engagement during the interview). One way to make accurate predictions in a way that is simultaneously motivationally congruent is therefore to define the behavior of interest strategically (Amemiya et al., 2023).

Numerous examples exist of individuals defining or re-defining criteria in a manner that suits their pre-existing motivations or biases (Munro et al., 2010; J. E. Phelan et al., 2008; Uhlmann & Cohen, 2005). Uhlmann and Cohen (2005), for example, found that participants displayed gender bias when assigning individuals to jobs, justifying their choices by redefining the criteria for success to be whatever their chosen candidate happened to have. Examples of such outcome re-definition are not limited to experiments, however. In fact, Roithmayr (2014) posits that the lengthy modern-day Harvard application that attempts to screen for qualities such as well-roundedness and leadership actually emerged in response to an influx of Jews into Harvard in the 1920s. Before this, according to Roithmayr, Harvard admissions were relatively straightforward, with applicants simply needing to pass a subject matter test to receive admissions. After an influx of Eastern European Jews, the university began to require much longer applications and to redefine their admissions criteria to favor participation in certain race- and class-coded activities, such as crew or tennis. In this way, the admission committees actually began to devalue academic excellence in favor of other criteria that allowed them to maintain the student body they wanted. She notes that this was not necessarily a conscious or intentional choice to specifically exclude Jews, but that the decision nevertheless served to shift the outcome of interest and in doing so favor White Anglo-Saxon men at the exclusion of others. Other examples of motivated outcome re-definition may include employers prioritizing abstract notions of “fit” to select White candidates over Black candidates (Liera & Ching, 2019; Sensoy & Angelo, 2017) or individuals defining crime as primarily street crime rather than corporate crime to maintain associations of Blackness with criminality.

## What Implications Does This Have for Individual Differences in Category Structures?

The pervasiveness of structures is such that structural effects on categorization are likely to play some role in most people’s social categorizations. However, this is not to say that these effects will be uniform across people; rather, the model suggests a number of places where individual differences are likely to emerge. Sewell’s notion of agency is useful here: drawing on previous conceptualizations, he proposes that individual actors have agency, which leads to the capacity to maintain and/or transform structures (Sewell, 1992). In particular, actors can transpose and apply schemas to new contexts, and can reinterpret or mobilize resources in terms of different schemas. However, this agency is not uniform; Sewell argues that both the kind and extent of agency vary greatly across individuals. People may thus vary both in the schemas and resources they have access to, as well as in factors that shape the centrality of these schemas and resources to their cognitive processing and worldviews.

The model proposed here, in which the selection of category dimensions on the individual level is shaped by structurally produced motivations, therefore suggests a number of individual differences that might emerge in category structures. This model suggests that categorization is shaped by a weighted loss function, such that perceivers are motivated not only by accuracy but also by schema stability and the material benefits that arise from a particular way of categorizing. The “weight” assigned to each of these contributors may differ from person to person; material benefits may influence some perceivers’ category structures to a greater degree than others, for example.

One obvious source of differences arises from what material benefits the perceiver actually stands to gain as a function of their group membership. People in dominant groups who benefit from the social structure are likely to be more motivated to maintain the current shared schemas than the people in oppressed groups who are harmed by these schemas (e.g., Cech & Blair-Loy, 2010). As a result, dominant group members are less likely to be aware of structural sources of inequality, including shared schemas. White people, for example, perceive less racism in both isolated incidents and systemic manifestations of racism than do Black people, due to Whites’ lower historical knowledge (Nelson et al., 2013) and resulting at least in part from identity-maintaining motivations (Adams et al., 2006; Kurtiş et al., 2010; Unzueta & Lowery, 2008). Furthermore, since the predominant schemas in a society are generally positive toward dominant groups, structural motivations may better align with individual or group-based motivations for members of these groups. For someone in a dominant group, adopting shared schemas is likely to also produce a positive image of the ingroup, whereas group-based self-esteem and shared schemas may be more opposed for someone in a marginalized group.

In many instances, the story is not so simple, however; in the context of race, for example, different racial groups outside of the Black/White binary have a more complicated relationship with oppression, with a single group both benefiting in some ways and being harmed in other ways by dominant racial schemas. In Canada and the United States, for example, many East and South Asian racial groups have been the subject of seemingly positive “model minority” stereotypes, which position them as relatively smart, hard-working, and economically successful. Despite the apparent increase in status such stereotypes might be expected to afford, the model minority myth has resulted in increased standards and a dismissal of the racism experienced by Asian Americans, while also functioning to maintain racial hierarchies by placing the onus on members of minority groups to improve their lot (Kiang et al., 2017; J. H. J. Kim et al., 2021; Shih et al., 2019; Wong & Halgin, 2006). The types of schema-maintaining or schema-dismantling motivations that emerge in response to such stereotypes will therefore be an important factor in predicting the attentional allocations of such perceivers.

In many cases, even those who are being harmed by current societal systems may nevertheless be motivated to maintain those systems (Jost & Banaji, 1994). Even when alternative systems would produce a more just distribution of material outcomes, the perceived costs of changing current systems may be sufficient to induce system-justifying motivations in members of disadvantaged groups, producing patterns of attentional allocations that mirror those of dominant group members. Thus, it is not simply the objective distribution of resources but the *perceptions* of both current distributions and the possible alternative distributions that drive motivations to maintain current societal structures. Although dominant schemas and categories may not always be beneficial on an immediate material level, they may compensate for material disadvantages through the increased reliability and predictability afforded by stable, persisting systems.

Dominance and power are determined by an intersection of dimensions, and an individual can be dominant along one dimension and marginalized along another (as in the case of a White gay person or a cisgender person of color). Although we suggest that those who occupy privileged positions along one or more dimensions will be motivated to categorize in a way that maintains current structural schemas, this is not to say that they will necessarily pay more attention to dimensions along which they are dominant. In fact, one of the primary ways that power maintains itself is through invisibility (McDermott & Samson, 2005; Phillips & Lowery, 2018); rather than being named as categories, dominant categories are often seen as the “default” mode of being, with departures from this default characterized as “other.” Thus, people in dominant groups may actually be motivated to draw explicit attention *away* from the dimensions that impart power to them, since doing so draws attention away from their privilege (Knowles et al., 2014; Phillips & Lowery, 2020).

The motivated denial or disregarding of one’s privilege may be especially prominent in the face of the backlash engendered by renewed public discourse on privilege and oppression. Although those in dominant groups will be motivated to categorize in a way that maintains structural schemas, this may take the form of explicitly down-weighting attention to dimensions they are dominant along. For example, members of dominant racial groups may endorse colorblind ideologies over more explicitly multicultural ones, since they view multicultural ideologies as threatening to their self-concepts (Rios, 2022). This down-weighting is a strategy for maintaining dominance, as drawing attention away from one’s privilege prevents it from being questioned, thereby entrenching it as the default. In this way, those in dominant groups can maintain the invisibility of their advantaged status. For example, Phillips and Lowery (2018) propose that White privilege is maintained through a kind of “herd invisibility,” in which individual White people are motivated to maintain their positive self-images or their group’s status, and so engage in behaviors to mask their privilege. The confluence of these individual behaviors results in societal-level invisibility of White privilege, which can protect the status of even those White people who do not engage in these behaviors.

In addition to differing opportunities for material benefits, perceivers may differ in their need for stable, shared schemas. Some individuals may be more willing to update their schemas, while others (such as those who are especially intolerant of uncertainty; Buhr & Dugas, 2002) may be more likely to cling to existing schemas rather than accommodating new information (Piaget, 1952). Indeed, some research has shown that individuals who are highly open to change have a higher social justice action orientation (Tittler et al., 2020)—in other words, are more likely to take actions that challenge dominant structural schemas. Paralleling this, other work has shown individual differences in the desire for shared reality, with conservatives demonstrating greater motivations to share reality with their ingroups than do liberals (Stern et al., 2014). These differences in the tendency to assimilate vs. accommodate information into structural schemas may represent a key source of individual variability in the extent to which the categories a perceiver uses conform to social structures.

Perceivers may also differ in the intermediary motivations that function to maintain schemas. Although motivations may be consistent on a very high level (i.e., to maintain structural schemas and get material benefits), the lower-order motivations that arise from these and actually drive behavior may vary from person to person. These different motivations, while having some important differences, are shaped by the same structures and hierarchies, and therefore can in turn reproduce these structures in similar ways. One person’s desire to maintain schemas may result in a strong belief in meritocracy: believing that those who are on top got there because they deserve it (and, paralleling that, those who are on the bottom are there because they deserve to be there) can be a powerful way to rationalize and justify inequalities (O’Brien et al., 2009; Phillips & Lowery, 2020), further reinforcing schemas and resource distributions. Another person’s schemas may instead result in a high intolerance for social uncertainty. This person may be reluctant to acknowledge the complex societal sources of inequality and may instead turn to simple answers. These simple answers, of course, are likely to be the ones provided by dominant schemas which are readily available and pervasive. A third person may have been raised in an environment in which rampant racism and sexism were perpetuated by their loved ones. This person may be unwilling to acknowledge and dismantle unjust structural schemas because doing so would challenge their pre-existing notions and require them to update their beliefs about the character of their loved ones for the worse. In this way, a variety of lower-order motivations may serve the function of maintaining schemas and resource distributions in different people.

Another source of variation may arise in perceivers’ awareness of societal schemas and willingness to oppose unjust ones. Members of dominant groups can react in a variety of ways to their privilege, such as by denying the existence of their privilege, distancing themselves from the privileged group, or working to dismantle systems of oppression (Knowles et al., 2014; Leach et al., 2009). Dismantling oppressive structures is difficult but not impossible; according to Sewell (1992), the reproduction of structures can happen without awareness but is not automatic. Rather, individuals have agency and can counteract this reproduction: the very agency that acts to maintain structures can also serve to transform them through the alteration of schemas and remobilization of resources. Transforming these structures, however, requires an awareness of the structures that are being reproduced, the knowledge of how to counteract these structures, and the willingness to do so despite the costs to oneself for failing to predict and be predictable. Thus, individuals who have a better understanding of these structures and are invested in dismantling them are likely to be less driven by schema maintenance and resource gain, and may even be driven to counteract dominant schemas and resource distributions. In fact, if the structural causes of inequality are made apparent, even young children will attempt to rectify this inequality, whereas the same is not true when inequality is attributed to merit differences (Rizzo et al., 2020). Social justice orientation or critical consciousness (Diemer et al., 2017) may be useful proxies for this awareness of and willingness to oppose unjust dominant schemas, with those with a greater commitment to social justice more willing and able to challenge structural schemas that perpetuate inequality.

Finally, structures and their influence are shaped by the wider cultural context that an individual is located within. In particular, culture may govern the creation of category-relevant schemas and the distribution of resources, as well as the way these structural factors interact with individual cognitions. Research has already demonstrated a potentially strong role for culture in governing patterns of attention, with “Easterners” adopting a more holistic attentional approach that focuses more on the relations among things, while “Westerners” tend to concentrate their attention more on the focal objects of a scene (Boduroglu et al., 2009; Cohn et al., 2012; Kuwabara & Smith, 2012; Nisbett & Masuda, 2003; Nisbett & Miyamoto, 2005). Although speculative at this time, there may similarly be a role for culture in shaping the allocation of attention to social category dimensions, with cultural factors influencing people’s motivations for shared schemas and resources. For example, it is possible that those in collectivistic cultures where interdependent self-construals are more common (Markus & Kitayama, 1991) may place greater weight on shared schemas, as maintaining a shared reality with others is more central to their self-concept. Those in “tighter” cultures, which have strong societal norms and discourage deviance from those norms (Uz, 2015), may similarly place greater weight on shared reality, as social cohesion in such a society would especially depend on maintaining the reality underlying those norms. Finally, those in more hierarchical societies, where the difference between the richest and poorest members is very stark, may be particularly oriented toward gaining and maintaining resources.

## How Does This Fit With the Idea of a “Rational Actor” or the Utility of Social Categorization as Information Reduction?

This work has a number of implications for the idea of the social perceiver as a “rational actor” or the utility of social categorization as information reduction, and in particular suggests a number of new ways of thinking about accuracy in social categorization. First, this work suggests that structural motives *constrain* accuracy motives. Under the idea that social perceivers are motivated to maintain shared schemas that confer material benefits, the problem of information reduction is changed. The problem for a social perceiver to solve becomes not just how to efficiently condense information in a way that allows for relatively accurate decisions while maintaining cognitive resources, but how to do so in a way that will line up with how others are condensing information to allow for shared schemas. In essence, the problem to be optimized becomes a weighted minimization of accuracy with other schema-relevant motivations. This idea fits with the argument made by Howard (1994), who suggests that the common assumption of cognitive efficiency in the domain of stereotyping is problematic since information is created and held inter-individually rather than individually, such that a single individual’s cognitive limits are less relevant.

Second, structural motives can *shape the definition* of “accuracy.” Social information is largely ambiguous, allowing for multiple interpretations that may be equally valid or “accurate.” In these cases, people may tend toward interpretations that fit within societal narratives both because they are most readily accessible and because they allow the perceiver to simultaneously reach the goals of accuracy and structural maintenance. Most social decisions or judgments occur at a relatively abstract level (e.g., “will this person make a good friend” or “who will be the best candidate for the job”), but the information we use to arrive at those decisions is much more concrete (e.g., “he often shows up late to events” or “this candidate scored in the 80th percentile on this standardized test of analytical reasoning”). Thus, there are many ways for abstract questions to be translated into specific indices, and often not a clear “best” conceptualization of a question, leaving a lot of room for ambiguity and bias. As a result, the way accuracy itself is measured and defined may be subject to motivational influences. In the simplest case, if accurate social perception is about the similarity between predicted behaviors and actual behaviors, a perceiver can increase accuracy either by changing their predictions to line up with reality or by changing the behaviors they see to line up with their predictions, through selective (mis)interpretation of behaviors or by shifting their focus to different kinds of behaviors. This may be especially likely to occur in more abstract domains since there is more latitude for defining the behavior of interest. Furthermore, these higher-level definitions are not directly observable or interpretable and can therefore be difficult to challenge or correct (both by the self and by others), since corrective feedback is not provided in the way that it might be at lower, more concrete levels. For example, there can be a lot more reasonable disagreement about the abstract statement “that person is a criminal” than the more concrete statement “that person just punched someone in the face.” In other words, prediction errors at lower (more concrete) levels may result in updating of low-level beliefs, but may be insufficient to update high-level or global schemas, since altering these schemas has the potential to catastrophically disrupt one’s shared global framework for making sense of the world (Wheeler et al., 2020). This is not to say that social reality is completely neglected, but simply that the perceiver’s situation-specific motivations (including accuracy) are all localized within societal-level schemas that constrain and shape both what those specific motivations are and how they influence categorization.

Finally, structures can *create* accuracy. Because structures are comprised of both resources and the schemas that we use to make sense of them, social perception is subject to a third variable problem. In particular, shared schemas can fit with the goal of maximizing a perceiver’s utility within a situation, but this is because utility itself is in large part shaped by the resource distributions to which the schemas are tied, and not because there is necessarily any kind of “ground truth” to them. For example, a perceiver who is trying to predict crime rates using demographic information will probably have high accuracy if they use targets’ race and class as predictors. However, the very reason that race and class are salient to the perceiver in the first place is because they follow the resource distributions that are tied to crime. In other words, the same variable (resource distributions) is producing both the cognitive schemas and the behaviors these schemas are trying to predict. These resource distributions both (partially) create the schemas and cause these dimensions to be particularly salient. Critically, making use of this information is still not a value-neutral tracking of statistical distributions because of the two-way nature of this relationship: upholding these schemas causes the further maintenance of resource distributions. As Howard (1994) put it, “social categorizations not only contribute to, but also reflect, social structures” (p. 212).

## What Implications Does This Have for Reducing Prejudice and Dismantling Structures?

A model in which social categorizations are produced by and contribute to social structures has a number of implications for how best to reduce harmful category use and dismantle structures. If structures are comprised of shared schemas that are underpinned and reflected in resources, attempts to dismantle structures could proceed in a number of ways: by intervening on the resource level, by intervening on shared schemas, or by intervening on the link between schemas and resources. Psychology, with its focus on individual cognitive processes, is not as well equipped to target resource distributions, which instead requires change at the level of the system. It is, however, equipped to act on shared schemas and to attempt to partially sever the link between schemas and resources. On the schema level, if social categorizations both contribute to and reflect social structures (Howard, 1994), deliberately changing the way we categorize may be one way to change (or at least avoid reinforcing) social structures. An awareness of how social categories are constructed and reified, as well as how the “obvious” or “default” ways of categorizing may in fact be the product of shared structural schemas, can help in identifying these structures for what they are and eventually dismantling them.

Psychological research is also well-positioned to aid in severing the link between shared schemas and resources. Since structural schemas need to be underpinned and reflected in resources to be maintained, people who are motivated toward maintaining structural schemas will not only selectively update cognitive structures through attention but will also take actions to ensure that resource distributions are maintained. For example, stereotypes that prevent hiring managers from choosing a Black job candidate over a White one function to perpetuate the resource distributions that underlie the schema (i.e., by giving a high-paying job to a White person over a Black person). Interventions that sever the link between schemas and resources can therefore be effective in reducing this cycle. For example, policies like blind resume review or reconsidering the use of standardized testing do not necessarily change the schemas that position Black job applicants as inferior, but they do help ensure that these schemas do not further entrench existing resource distributions. In the absence of such policies, schemas can bias application review such that Black applicants are disproportionately rejected from high-paying jobs. The implementation of these policies can instead reduce the impact of schemas on resources, such that resources can become more evenly distributed (with the hope that this can eventually result in changed schemas as well). Of course, these individual-level interventions on their own are not enough to rectify decades of entrenched and self-reinforcing resource distributions and must be accompanied with system-level changes in the longer term.

Although we have focused our discussion on individual social perceivers who are forming judgments and decisions of other people, we believe many of the principles we have discussed can also be applied to the way researchers think about social categories in the context of their research. Over the past few decades, psychology has come under criticism for its treatment of race as a given rather than interrogating the practices and systems that create race as a category (Hollander & Howard, 2000; Hopkins et al., 1997; Leach, 2002; Martinez & Paluck, 2020; Reicher & Hopkins, 2001; Smedley & Smedley, 2005). A number of these criticisms argue that race as a variable cannot be causal and must be interpreted in light of its associations with racism (Sen & Wasow, 2016; Zuberi, 2000). By recognizing the continuous nature of dimensions such as race and gender, psychologists can provide important insight into how perceivers actively create and perpetuate the categorical aspects of these dimensions. We echo Hopkins and colleagues (1997), who argued that “as ‘race’ is not a natural category but rather racialized categories are socially constructed, we need a social psychology which focuses upon the social processes and strategies through which categories are constructed (and racialized)” (p. 312).

Furthermore, if structures determine which dimensions we attend to, we should be cognizant as researchers of how our own schemas and motivations influence the questions we ask (e.g., “are stereotypes accurate?” vs. “what function do stereotypes serve?”) and the categories we choose to focus on (e.g., Black, White, and Hispanic as racial groups), as well as of how these questions and categories in turn reinforce the structures that produce them. In conducting research on social categories, we render certain dimensions relevant simply by choosing to study them; reflection on the dimensions that we have given importance and those that we have neglected is therefore key. For example, looking for differences between men and women and treating “gender” as the causal variable for any variance may sometimes neglect the multitude of processes that create our categories of gender. The stimuli we select and present to participants also function as a signal about what dimensions are worth attending to in the local context. A study with only Black and White male faces readily signals that race is the focal dimension, whereas a stimulus set that is more reflective of the local environment may present a more externally valid context for studying social perception. Similarly, psychologists should take care that the desire for shared schemas within the field does not lead to simplistic or narrow definitions of complex concepts. For example, characterizing stereotypes simply as any beliefs about a group, or prejudice as any feeling toward a group, leads to precise definitions that may nevertheless be divorced from the way these concepts are classically understood and used outside of the field (Vrantsidis & Cunningham, forthcoming). Instead, we can draw more heavily on research traditions (both within and outside of psychology) that aim to bring notions of power and dominance to our understanding of these concepts, in a way that both acknowledges the material implications of these topics and is not overly attached to existing schemas.

## Positionality Statement

Our thinking about the ways in which social structures interface with cognitive processes is necessarily informed by our own locations within these structures. First, living and working in Canada has shaped our intuitions on social identities (and thus many of the examples we draw upon)—specifically through the current and historical systems of race and gender relations in Canada and the United States. Although we believe that these principles will generalize, we are open to the fact that these intuitions may not fully characterize other important factors, and additional cultural forces may need to be considered. Second, our approach to conceptualizing social categories and identities is embedded within the perspectives of experimental social-cognitive psychology. We acknowledge that although we have worked to expand the scope of inquiry to include additional academic perspectives, our focus and training concern individual cognitive representations situated in social contexts. Our perspective alone, therefore, cannot articulate all levels of analysis, and complementary approaches are needed to more fully answer these questions. Finally, our approach and assumptions are naturally informed consciously or unconsciously by our experiences, identities, ideologies, and demographics, including our positions within the very hierarchies and narratives we discuss in this paper.

## Notes

1. However, our view on the role of accuracy in these processes (“How Does This Fit With the Idea of a ‘Rational Actor’ or the Utility of Social Categorization as Information Reduction?” section) departs from that of the affordance management approach.
